# Event-Based Optical Flow Estimation with Spatio-Temporal Backpropagation Trained Spiking Neural Network

**DOI:** 10.3390/mi14010203

**Published:** 2023-01-13

**Authors:** Yisa Zhang, Hengyi Lv, Yuchen Zhao, Yang Feng, Hailong Liu, Guoling Bi

**Affiliations:** 1 Changchun Institute of Optics, Fine Mechanics and Physics, Chinese Academy of Sciences, Changchun 130033, China; 2 College of Materials Science and Opto-Electronic Technology, University of Chinese Academy of Sciences, Beijing 100049, China

**Keywords:** event camera, optical flow estimation, spiking neural network, spatio-temporal backpropagation

## Abstract

The advantages of an event camera, such as low power consumption, large dynamic range, and low data redundancy, enable it to shine in extreme environments where traditional image sensors are not competent, especially in high-speed moving target capture and extreme lighting conditions. Optical flow reflects the target’s movement information, and the target’s detailed movement can be obtained using the event camera’s optical flow information. However, the existing neural network methods for optical flow prediction of event cameras has the problems of extensive computation and high energy consumption in hardware implementation. The spike neural network has spatiotemporal coding characteristics, so it can be compatible with the spatiotemporal data of an event camera. Moreover, the sparse coding characteristic of the spike neural network makes it run with ultra-low power consumption on neuromorphic hardware. However, because of the algorithmic and training complexity, the spike neural network has not been applied in the prediction of the optical flow for the event camera. For this case, this paper proposes an end-to-end spike neural network to predict the optical flow of the discrete spatiotemporal data stream for the event camera. The network is trained with the spatio-temporal backpropagation method in a self-supervised way, which fully combines the spatiotemporal characteristics of the event camera while improving the network performance. Compared with the existing methods on the public dataset, the experimental results show that the method proposed in this paper is equivalent to the best existing methods in terms of optical flow prediction accuracy, and it can save 99% more power consumption than the existing algorithm, which is greatly beneficial to the hardware implementation of the event camera optical flow prediction., laying the groundwork for future low-power hardware implementation of optical flow prediction for event cameras.

## 1. Introduction

The estimation of motion patterns corresponding to spatio-temporal variations of structured illumination commonly referred to as optical flow, provides vital information for estimating ego-motion and perceiving the environment [1]. In the past years, the main optical flow estimation technologies have been proposed for traditional frame-based image sensors. They directly use optical flow algorithms to process the light intensity information in the scene obtained by the image sensor with a fixed exposure time while ignoring the dynamic information in the scene. Although the existing optical flow estimation technology is sufficient for machine vision based on the frame-based image sensor, the frame-based image sensor suffers from issues such as the inability to obtain the target information clearly under extreme lighting conditions, motion blur during high-speed motion, high power consumption, low information value density, etc.

**Figure 1:**
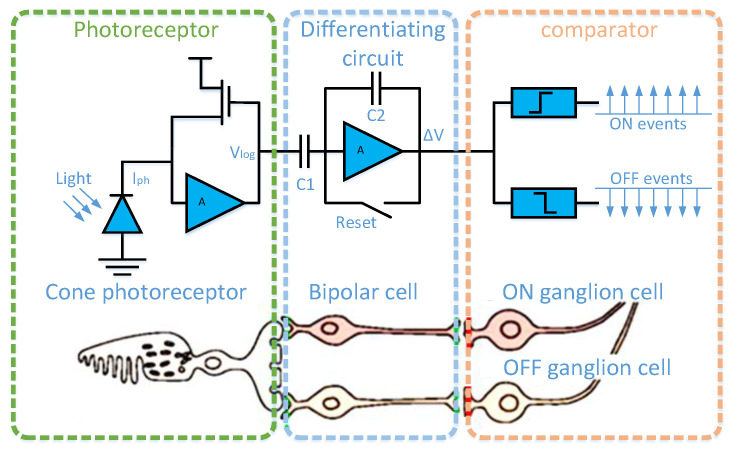
Three-layer model of a human retina and corresponding event camera pixel circuitry. The first layer is similar to retinal cone cells for photoelectric conversion; the second layer, similar to bipolar cells in the retina, is used to obtain changes in light intensity; the third layer is similar to the ganglion cells of the retina for outputting the light intensity change sign.

**Figure 2:**
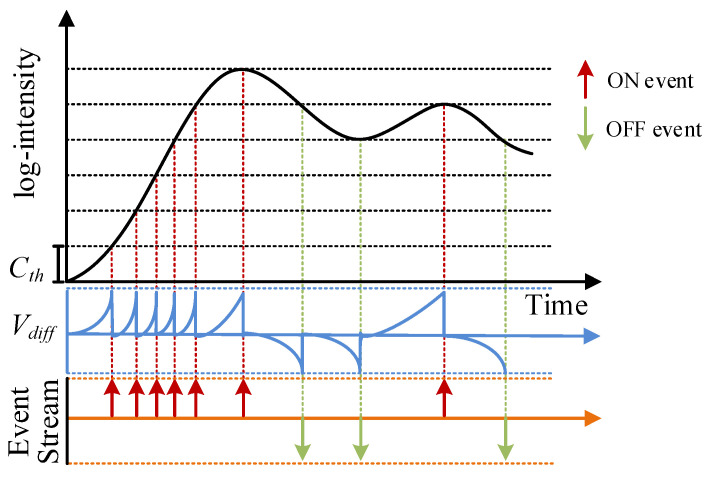
Schematic diagram of the event camera event generation process: when $\left|V_{diff}\right|>C_{th}$, an ON or OFF event is generated.

Therefore, the event camera emerged as the times required, and is also called a dynamic vision sensor, bio-inspired sensor, or neurological sensor [2,3,4,5]. The dynamic vision sensor is inspired by the biological retina. As a result of its unique pixel structure (Figure 1), it only responds to places where the light intensity changes in the scene and has the strength of high dynamic range and low data redundancy [6]. The output signal is called event $e_{i}=e\left(x_{i},y_{i},t_{i},p_{i}\right)$, which contains position, microsecond timestamp, and polarity information. For the event camera data generation process shown in Figure 2, when the light intensity changes, the $V_{diff}$ perceived by the pixel at time *t* is greater than the event trigger threshold $C_{th}$, generating an event.

, an ON or OFF event is generated.

Combined with the characteristics of the event camera, The set of output events in the spatiotemporal domain is known as the spatiotemporal event stream. The spatiotemporal event stream can be defined as the following [7].

(1) $$E=\overset{N}{\underset{i=1}{\sum }}e\left(x_{i},y_{i},t_{i},p_{i}\right)$$

where e is an event of the spatiotemporal event stream, $\left[x,\;y\right]$ denotes location of the pixel generating the event, $p\;\in \;\left\{-1,+1\right\}$ indicates the polarity of the change in illumination at the pixel causing the event, and 𝑡 represents the time at which the event occurred; i is the index of events in the spatiotemporal event stream, and Σ indicates adding the new event to the spatiotemporal data stream.

High temporal resolution (in the unit of microseconds), low power consumption, and high dynamic range compared with frame-based cameras make event cameras suitable for estimating high-speed and low-light visual motion in an energy-efficient manner. Hence, event cameras have been gradually applied to object tracking [8,9], surveillance and monitoring [10,11], star tracking [12], etc.

Event cameras are also suitable for optical flow estimation since the precise timestamp at pixel-level intensity changes directly encodes fine-grain motion information. However, the event camera represents a paradigm shift in computer vision because of its principle of operation and unconventional output [13]. Because of its unique data format, the existing optical flow estimation algorithm is unsuitable for event cameras. Therefore, a new algorithm is urgently needed to promote the application of the event camera.

### 1.1. The Related Work to Predict Optical Flow for Event Camera

In recent years, many researchers have proposed optical flow estimation based on event cameras to promote the application of event cameras in machine vision. According to the working principle of their algorithms, these optical flow estimation methods can be roughly divided into two categories. One is the optical flow estimation algorithm based on traditional methods. The other is the modified version of the optical flow estimation algorithm based on the neural network.

In the basic algorithm of event camera optical estimation, the gradient method, plane fitting method, and frequency method have all achieved perfect optical flow estimation results. Benosman, R. and Brosch, T. used the gradient-based Lucas–Kanade algorithm to estimate the optical flow of discrete spatiotemporal data [14,15]. The method proposed in [16,17] extracts optical flow by calculating the spatiotemporal surface gradient of events using the local plane fitting method. The bio-inspired method proposed in [18] is more suitable for hardware implementation. Moreover, the correlation-based method proposed in [19,20] uses a convex optimization algorithm to process event groups to predict the optical flow. In addition, Liu, M. interestingly uses an adaptive block matching technique to estimate sparse optical flow [21].

For neural network algorithms for the event camera’s optical flow estimation, supervised training, self-supervised training, and unsupervised training are all applied to the event camera’s optical flow estimation by researchers. EV-FlowNet [22] proposed a self-supervised training method to train the traditional convolutional neural network (CNN) network to estimate the optical flow for the event camera. The input to the network consists of the per-pixel last timestamp and count of events over a specific time window. Then, the gray image is used to replace the ground truth for self-supervised training for the network. Similarly, the optical flow prediction net using the gray image in the self-supervised way is also present in [1,23]. On the contrary, Zhu, A.Z. proposed a “voxel grid” event representation method to retain the time information of the event camera, and used motion compensation to calculate the loss function for unsupervised training of the network [24]. Gehrig, M. used the “voxel grid” for event representation and then used ground truth for supervised training of RAFT network architecture [25].

Although the above-mentioned neural network method has made optical flow prediction on the spatiotemporal data of the event camera possible, most of the methods mentioned above slice the spatiotemporal data stream according to a fixed number of events or a fixed time interval and then convert it into image frames, sending the image frames into the traditional convolutional neural network for training. The spatiotemporal data stream is sliced by the fixed number of events or the fixed time interval. Then, the event slice is converted into image frames and sent into the traditional convolutional neural network for training. However, the method of slicing with a fixed number of events or a fixed time interval and then converting to an image frame will result in motion blur or information loss in the image frame. Moreover, the network will calculate every pixel in the image frame, significantly increasing the amount of calculation and losing the advantage of the low data volume of the event camera.

### 1.2. The Main Contributions of This Paper

This paper also uses the learning method to predict optical flow, but it is different from the above methods. First, this paper uses the method in [7] to adaptively slice the spatiotemporal data stream and then sends the event slice into the trained spike neural network in discrete form for optical flow prediction. The slice has no information loss or motion blur, which improves the optical flow prediction accuracy. Then, to better process the discrete spatiotemporal data stream output by the event camera, we propose an end-to-end spike neural network model, which can receive the discrete spatiotemporal data stream, dramatically reduce the network computation, and retains the advantage of the low data volume of the event camera. In addition, in order to solve the problem that high-quality ground truth is not easy to obtain, we use the gray image to calculate the loss function to train the spike neural network in a self-supervised manner. Finally, we test on the public dataset to verify the advantages of our method.

In a word, the main contributions of this paper are as follows:

1. Build a spike neural network architecture that is more suitable for discrete spatiotemporal data stream so that it can directly process discrete spatiotemporal data of the event camera, reduce the amount of computation, and retain the advantage of the low data volume of event cameras;
2. Aiming at the problem that the existing training methods of the spike neural network mainly focus on the spatial domain but pay less attention to the time domain, the network is trained using high-performance spatial temporal backpropagation combined with the spatiotemporal information of the event camera to improve the accuracy of optical flow prediction.

## 2. Materials and Methods

### 2.1. Spiking Input Event Representation

Because the output data of the event camera is an asynchronous and discrete spatiotemporal data stream, which is not compatible with the existing convolutional neural network model, many researchers construct spatiotemporal data into image frames with various methods and then use existing convolutional neural networks to predict the optical flow.

**Figure 3:**
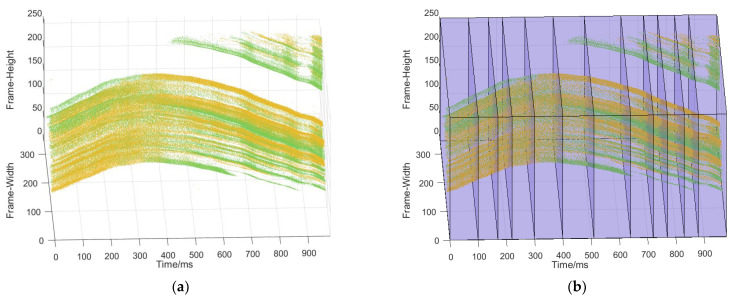
(**a**) spatiotemporal data stream of the event camera; (**b**) spiking input event representation obtained by [7].

The most commonly used representation is multiple discretized frames of event counts [1,23,24,26,27], the per-pixel average, or the most recent event timestamps [22,28,29]. However, this method of constructing “frames” from event slices can lose the advantages of event cameras in terms of high time resolution and low data volume. In addition, [24] proposed a discretized event volume that deals with the time domain as a channel to retain the spatiotemporal event distributions. However, the number of input channels increases significantly as the time dimensions are finely discretized, further aggravating the computation and parameter overheads [1]. Gehrig, D. proposed an event representation method that integrates all information [30] which can compress a certain dimension of the event to obtain the existing image frame, voxel and other event representation methods. However, this method still needs to preprocess the spatiotemporal data stream and does not enable the network to process the discrete spatiotemporal event stream directly. To directly use the discrete characteristics of spatiotemporal data and reduce the computational complexity of the network, we use the spike neural network to directly process the spatiotemporal data. At the same time, in order to avoid target loss or motion blur in the event slice for optical flow estimation, we use the adaptive slicing method to slice the spatiotemporal data stream [7]. Then, event slices are sent into the network in discrete form, as shown in Figure 3. Therefore, the events sent to the network can be expressed as:

(2) $$E\left(∆t\right)=\overset{t_{k}+∆t}{\underset{t_{k}}{\sum }}\{e\left(x_{i},y_{i},t_{i},p_{i}\right)|t_{i}\in \left[t_{k},t_{k}+∆t\right]\}$$

where $t_{k}$ is the start of the event slice, ∆t represents the time length of the event slice, and $E\left(∆t\right)$ represents the event slice.

### 2.2. Spiking Neuron Models

Spike neural network is a new generation of artificial neural network model inspired by biology, which has strong spatiotemporal information representation and asynchronous event information processing capabilities. Spike neural network has many neuron representation models, but leaky-integrate-and-fire (LIF) is the most widely used model, which can be simply expressed by the following formula:

(3) $$\tau \frac{du\left(t\right)}{dt}=-u\left(t\right)+I\left(t\right)$$

where $u\left(t\right)$ is the neuron membrane potential at time t, τ is a time constant, and $I\left(t\right)$ represents presynaptic input determined by preneuronal activity and synaptic weight.

When the membrane potential $u\left(t\right)$ exceeds the given threshold $V_{th}$, the neuron will trigger a pulse and reset its potential to $u_{rest}$.

Wu, Y. optimized the traditional LIF and built an iterative LIF mode [31]. When we solve Equation (3), we obtain:

(4) $$u\left(t\right)=u\left(t_{i-1}\right)e^{\frac{t_{i-1}-t}{\tau }}+I\left(t\right)$$

Equation (4) shows that the membrane potential at time t is related to the membrane potential at $t_{i-1}$ and presynaptic input $I\left(t\right)$. Before the neuron receives a new input, the membrane potential decreases exponentially. When $u\left(t\right)>V_{th}$, the neuron sends out a spike, $u\left(t\right)$ is reset to $u_{rest}$, and then a new round of update starts. It can be seen that whether neurons generate spikes depends on the accumulation of presynaptic input in the spatial domain and the decay of membrane potential in the temporal domain.

The presynaptic inputs are accumulated spikes from other neurons at the last layer. Therefore, $I\left(t\right)$ can be represented by:

(5) $$x_{i}^{t,n}=\overset{l\;\left(n-1\right)}{\underset{j=1}{\sum }}w_{ij}^{n}o_{j}^{t,n-1}$$

where $w_{ij}^{n}$ is the synaptic weight from the *j*-th neuron in the pre-layer (n−1) to the *i*-th neuron in the post-layer (n) and $o_{j}^{t,n-1}$ is the output of the *j*-th neuron in pre-layer (n−1). when $o_{j}=1$, a spike will be sent. when $o_{j}=0$, the *j*-th neuron does not send a spike, and can be expressed as:

(6) $$o_{j}^{t+1,n}=\left\{\begin{array}{c} 1\;\;\;\;\;\;\;\;\;\;\;\;\;\;\;\;if\;u_{i}^{t+1,n}>V_{th}\\ 0\;\;\;\;\;\;\;\;\;\;\;\;\;\;\;\;\;\;\;\;\;\;\;otherwise \end{array}\right.$$

Therefore, Equation (4) can be changed to:

(7) $$u\left(t\right)=u\left(t_{i-1}\right)e^{\frac{t_{i-1}-t}{\tau }}+x_{i}^{t,n}$$

Next, we combine the fire reset mechanism in Equation (7). Considering the accumulation of the presynaptic input in the spatial domain and the decay of the membrane potential in the time domain, we iteratively update the LIF model to obtain the membrane potential of the *i-th* neuron in the layer(*n*) at time t:

(8) $$u_{i}^{t,n}=u_{i}^{t-1,n}f(o_{j}^{t-1,n})+x_{i}^{t,n}$$

(9) $$f\left(x\right)=\tau e^{-\frac{x}{\tau }}$$

**Figure 4:**
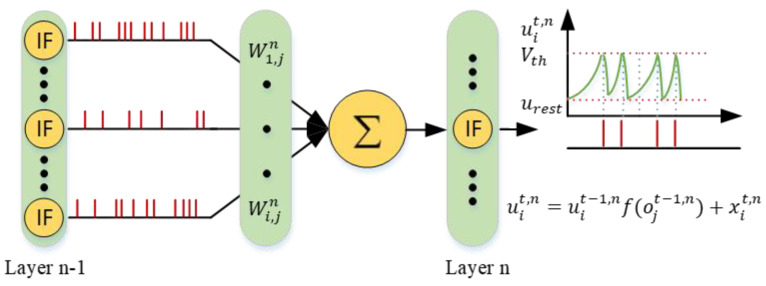
The update of membrane potential according to Equations (4)−(9).

Equation (8) is the iterative LIF model that is more consistent with the firing activity of the neuron. The model of spike iteration is shown in Figure 4. The iterative LIF model enables forward and backward propagation to be implemented on both spatial and temporal dimensions, which makes it friendly to general machine learning programming frameworks.

### 2.3. Network Architecture

**Figure 5:**
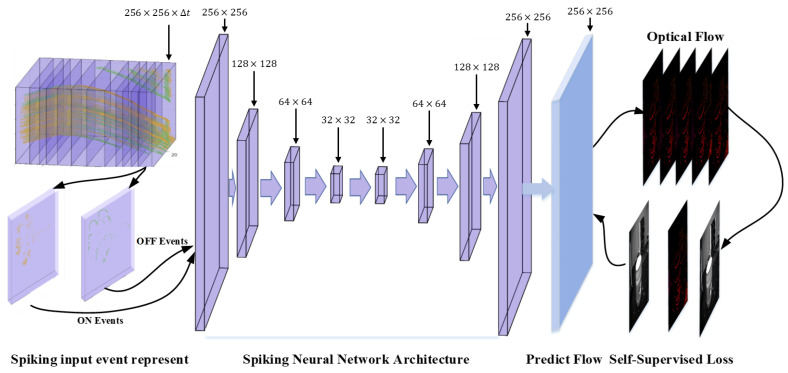
Spike neural network architecture for the event camera optical flow prediction. The events in the adaptive slice are divided into ON/OFF channels to enter the pyramid structure of the spike neural network in a discrete form, and the gray image is used to carry out spatio-temporal backpropagation in a self-supervised manner.

Our network architecture is similar to that in SpikeMS [32]. The end-to-end spike neural network model includes four spike feature coding layers and four spike feature decoding layers, as illustrated in Figure 5. The discrete spatiotemporal data (256×256×∆t) in the adaptive slice is divided into ON/OFF channels (2×256×256×∆t) and passes through the spike feature coding layer of the four pyramid structures (L1:256×256, L2:128×128, L3:64×64, L4:32×32) in a discrete form to complete the feature extraction. After that, four spike feature decoding layers (L5:32×32, L6:64×64, L7:128×128, L8:256×256) are sequentially used to complete the up-sampling. Finally, the optical flow prediction layer (256×256) outputs optical flow information with two channels.

### 2.4. Self-Supervised Learning of Optical Flow via Gray Image

Compared with the traditional optical flow datasets based on frame-based cameras, the number of optical flow datasets with ground truth that can be used for optical flow estimation of event cameras is relatively small. Therefore, we use a self-supervised learning method, which uses the gray image generated together with the asynchronous event stream of the event camera to train the spike neural network [22]. We combine the optical reconstruction loss ($L_{photo}$) and smooth loss ($L_{smooth}$) as the loss function of the network.

(10) $$L_{total}=L_{photo}+\lambda L_{smooth}$$

where *λ* is the weight factor.

We send the event slice $E\left(∆t\right)=\overset{t_{k}+∆t}{\underset{t_{k}}{\sum }}\{e\left(x_{i},y_{i},t_{i},p_{i}\right)|t_{i}\in \left[t_{k},t_{k}+∆t\right]\}$ and a pair of gray images ($F_{t},F_{t+∆t}$) generated in the corresponding time period into the network together to calculate the loss function.

The photometric reconstruction loss ($L_{photo}$) uses the light intensity consistency assumption. That is, the image obtained by mapping the first gray image $F_{t}$ with the estimated optical flow should be consistent with the second gray image $F_{t+∆t}$. The $L_{photo}$ is committed to minimizing the discrepancy between the second grayscale image and the mapped first grayscale image. The photometric reconstruction loss calculation method is as follows:

(11) $$L_{photo}\left(u,v;I_{t},I_{t+∆t}\right)=\underset{x,y}{\sum }\rho \left(I_{t}\left(x,y\right)-I_{t+∆t}\left(x+u\left(x,y\right),y+v\left(x,y\right),\right)\right)$$

(12) $$\rho \left(x\right)={\left(x^{2}+\eta^{2}\right)}^{r}$$

where $I_{t},I_{t+∆t}$ represents the light intensity of the first gray image and the second gray image, $u\left(x,y\right)$ and $v\left(x,y\right),$ representing the optical flow information in horizontal and vertical directions. ρ is Charbonnier loss, which is a generic loss used for outlier rejection in optical flow estimation [33].

The smoothness loss ($L_{smooth}$) enhances the spatial collinearity of the optical flow of neighboring pixels. That is, the optical flow of neighboring pixels should be consistent in direction. The $L_{smooth}$ is committed to reducing the optical flow difference between adjacent pixels, and then regularizing the optical flow. The $L_{smooth}$ calculation method is as follows:

(13) $$L_{smooth}\left(u,v\right)=\frac{1}{HW}\overset{H}{\underset{j}{\sum }}\overset{W}{\underset{i}{\sum }}\left(\left\|u_{i,j}-u_{i+1,j}\|+\|u_{i,j}-u_{i,j+1}\|+\|v_{i,j}-v_{i+1,j}\|+\|v_{i,j}-v_{i,j+1}\right\|\right)$$

where H is the height and W is the width of the predicted flow output. Therefore, the total loss is the sum of $L_{photo}\;$ and weighted $L_{smooth}$.

### 2.5. Spatio-Temporal Backpropagation

**Figure 6:**
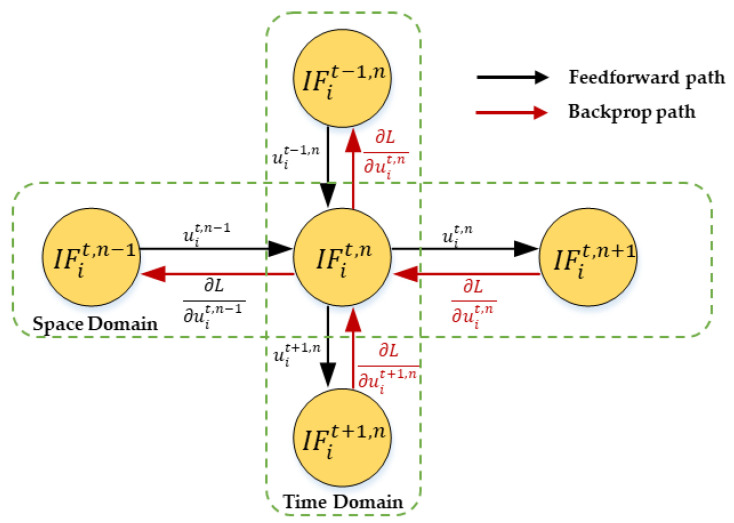
Spatio-temporal backpropagation in spike neural network. At the single-neuron level, the vertical path and horizontal path represent the loss L_total propagation in the space domain and time domain, respectively.

At present, direct supervised learning based on gradient descent theory and error backpropagation is used for the high-performance training of spike neural networks, but this method only considers the spatial information and ignores the dynamic information in the time domain. Therefore, many complex training techniques are needed to improve the network performance. When spatiotemporal data are propagated forward in the network, not only are the accumulation of the presynaptic space domain considered, but also use the decay of membrane potential in the time domain. Therefore, the backpropagation algorithm should be considered from two aspects: the space domain and the time domain. In order to make full use of the time characteristics of the spatiotemporal data of the event camera, and to reduce the complexity of network training and improve the network performance, we use spatio-temporal backpropagation [31] to effectively train the network. The backpropagation process is shown in Figure 6.

Considering the error propagation in the space and time domains, the chain rule of derivation can be used to obtain the following [34]:

(14) $$\frac{\partial L}{\partial o_{i}^{t,n}}=\overset{l\left(n+1\right)}{\underset{j=1}{\sum }}\frac{\partial L}{\partial o_{i}^{t,n+1}}\frac{\partial o_{i}^{t,n+1}}{\partial o_{i}^{t,n}}+\frac{\partial L}{\partial o_{i}^{t+1,n}}\frac{\partial o_{i}^{t+1,n}}{\partial o_{i}^{t,n}}$$

(15) $$\frac{\partial L}{\partial u_{i}^{t,n}}=\frac{\partial L}{\partial o_{i}^{t,n}}\frac{\partial o_{i}^{t,n}}{\partial u_{i}^{t,n}}+\frac{\partial L}{\partial o_{i}^{t+1,n}}\frac{\partial o_{i}^{t+1,n}}{\partial u_{i}^{t,n}}$$

In the process of backpropagation, we need to solve the presynaptic output $o_{j}^{t,n}$ derivative, but $o_{j}^{t,n}$ is a nondifferentiable Dirac function; therefore, we need to choose an appropriate function for $o_{j}^{t,n}$. To solve this problem, we use a rectangular function to approximate the reciprocal of spike activity [35]:

(16) $$h\left(u\right)=\frac{1}{a}sign(\left|u_{i}^{t,n}-V_{th}\right|<\frac{a}{2})$$

when $a\to 0^{+}$:

(17) $$\frac{\partial o_{j}^{t,n}}{\partial u_{i}^{t,n}}=h\left(u\right)$$

where *a* is the width of rectangular function.

### 2.6. Dataset

We use the public dataset Multi Vehicle Stereo Event Camera dataset (MVSEC) [36] to train and test our model. The MVSEC dataset includes two scenes: one is to carry the binocular event camera on a UAV to take pictures indoors, and the other is to carry the binocular event camera on a vehicle to collect data on the city streets. The dataset contains the attitude information and depth information of the event camera. In order to generate labeled event camera optical flow data for training and testing the event camera optical flow prediction network, Zhu, A.Z. used the attitude information and depth information in the dataset to generate the ground truth optical flow for the indoor flying, and outdoor day and outdoor light sequences. The gray images with timestamp information are also included in the dataset, so that we can use them for self-supervised training of the network. In order to make a fair comparison with the previous work [1,22,24,37], we only use the outdoor day2 to train our network. Indoor flying1, indoor flying2, indoor flying3, and outdoor day1 sequences are for evaluation only.

## 3. Experiment

### 3.1. Train Detail

We use the outdoor day2 in the MVSEC dataset to train the network model. There are two types of ground truths of optical flow in the dataset, one is generated between the *N*-th and *N* + 1-th (dt = 1) gray images, and the other is generated between the *N-th* and *N +* 4-th (dt = 4) gray images. Since we use adaptive slicing, we only use the dataset of dt = 1 to train the network and conduct comparative experiments. When using the gray image for self-supervised training, there may be optical flow information predicted by multiple event slices between two gray images. Therefore, it is necessary to map the predicted optical flow of all event slices in the two gray image frames to ensure the accuracy of loss function calculation.

Our framework is implemented in PyTorch. We use the Adam optimizer [38] and a learning rate of 0.001, and train with a batch size of 8 for 100 epochs. The weight on the smoothness loss *λ* in Equation (10) is set to 0.5. For the Charbonnier loss (12), we set *α* to be 0.45 and η was set to be 1e-3 similar to [1,22]. The threshold of the IF neurons are set to 0.75 (dt = 1) in the SNN layers.

### 3.2. Performance and Comparison with Other Methods

#### 3.2.1. Evaluation Index

In this paper, Average End point Error (AEE), which is commonly used in the field of optical flow prediction, is selected as the evaluation index of optical flow quality predicted by different methods. AEE mainly calculates the average European distance between the predicted optical flow of each pixel and the ground truth. The AEE calculation equation is as follows:

(18) $$AEE=\frac{1}{m}\underset{m}{\sum }\|{\left(u,v\right)}_{pre}-{\left(u,v\right)}_{gt}\|{}_{2}$$

where *m* is the number of pixels with optical flow information, ${\left(u,v\right)}_{pre}$ is the predicted optical flow information, and ${\left(u,v\right)}_{gt}$ is the optical flow information of ground truth.

#### 3.2.2. Experience Result

Our method compares with [1,22,24,37] in indoor flying1, indoor flying2, indoor flying3, and outdoor day1. Our method is the same as [1,22,37] and uses gray images to conduct self-supervised training for networks. Ref. [24] uses the image quality after deblurring as a loss function to train the network. In the experimental comparison, we no longer recalculate the experimental indicators of other methods but directly accept the indicators in [37]. Since we use adaptive slicing, we only use the dataset of dt = 1 to train the network and conduct comparative experiments. Table 1 provides the AEE evaluation results compared with prior event camera-based optical flow estimation works. As seen from Table 1, since the SNN is far less mature in backpropagation than the CNN, the results of the SNN for the event camera’s flow prediction in this paper are not as good as those of the best CNN. Our method aims to explore a spike neural network that can predict the optical flow for the event camera and pave the way for further hardware implementation. Therefore, the experimental results are as expected.

**Figure 7:**
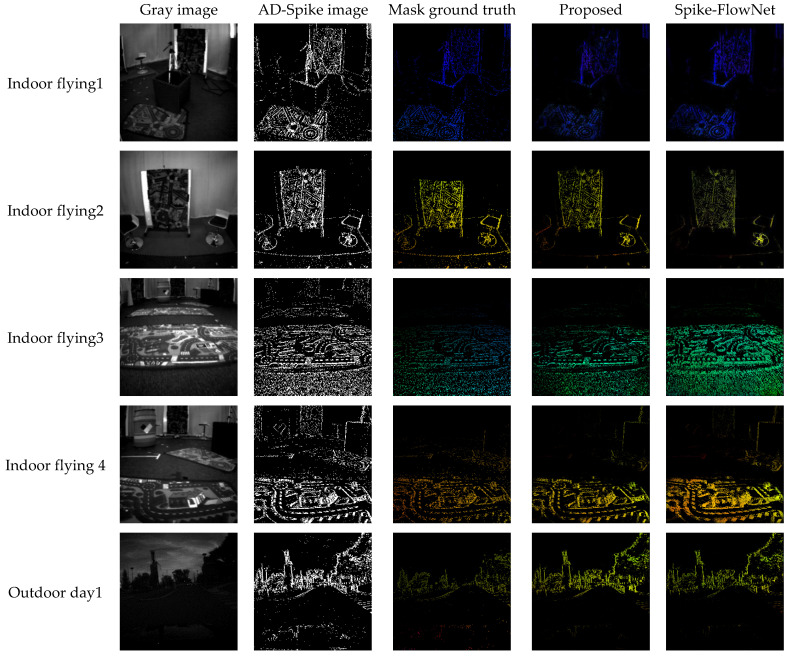
Optical flow comparison with Spike-FlowNet. From left to right: Grayscale image, spike image, masked ground truth, our masked predicted optical flow, and spike-FlowNet’s optical flow, where the images are taken from indoor flying1, indoor flying2, indoor flying3, and outdoor day1, respectively.

In [24,37], the do not disclose code and EV-FlowNet is not as good as Spike-FlowNet in index results, we only show Spike-FlowNet and our optical flow estimation results in Figure 7. All the optical flow in Figure 7 is basically a sparse optical flow computed at pixels at which events occurred. It is computed by masking the predicted optical flow with the spike image, where the images are taken from indoor flying1, indoor flying2, indoor flying3, and outdoor day1. The experimental results show that our method has a good information preservation effect at the edge of the target outline, and there is no motion blurring.

### 3.3. Ablation Studies

#### 3.3.1. Comparison for Networks

In order to verify that the spike neural network proposed in this paper can better handle the event camera’s discrete data and reduce the network’s computational load, in this section, we analyze the computational complexity of our approach in terms of the float point operations (*FLOPs*) and the theoretical power consumption between SNN and CNN with the same network architecture.

The calculation formula of *FLOPs* for the single-layer convolutional neural network when there is offset is [39]:

(19) $$FLOPs_{l}=\left[(C_{il}\times k_{wl}\times k_{hl}\right)+\left(C_{il}\times k_{wl}\times k_{hl}-1\right)+1]\times C_{ol}\times W_{l}\times H_{l}$$

where $C_{il}$ and $C_{ol}$ are the number of input and output channels of each layer of network, $k_{wl}$ and $k_{hl}$ the convolution kernel size of each layer, and $W_{l}$ and $H_{l}$ are the size of the feature map of each layer.

A “Multi-Add” is often regarded as a float point operation in computer vision papers. Therefore, the operation amount of the single-layer network is:

(20) $$FLOPs\_CNN_{l}=C_{il}\times k_{wl}\times k_{hl}\times C_{ol}\times W_{l}\times H_{l}$$

The number of floating point operations in the entire CNN network is:

(21) $$FLOPs\_CNN=\sum_{l}C_{il}\times k_{wl}\times k_{hl}\times C_{ol}\times W_{l}\times H_{l}$$

Because of the binary nature of spike events, SNN performs only an accumulation (AC) per synaptic operation. Compared with the addition operation (0.9 pJ), the power consumption of the multiply-accumulate operation (4.6 pJ) is 5.1 times that of the add operation [40]. Thus, in anticipation of deploying SNN on the neuromorphic chips, we demonstrate the power savings by comparing the number of operations by a metric proposed in [1]. Table 2 provides the average number of synaptic operations in SNN along with a conservative estimate of the energy benefit compared to a CNN. We can observe that SNN has a significantly lower number of synaptic operations and power compared with CNN.

#### 3.3.2. Comparison for Event Slicing Method

Before optical flow prediction, the event flow needs to be cut into event slices and then input into the optical flow prediction network in various forms. The existing event slicing methods will lead to motion blur or information loss of the target in the scene, affecting the accuracy of optical flow prediction. Therefore, in this experiment, only the event slicing method is changed, and optical flow prediction is conducted on the SNN proposed in this paper with the same dataset. For the convenience of comparison, we choose a fixed time window (dt = 1) as the slicing method for the comparison experiment. The experimental results are shown in Table 3. It can be seen from the experimental results that the adaptive slicing method used in this paper can better improve the optical flow prediction accuracy

**Figure 8:**
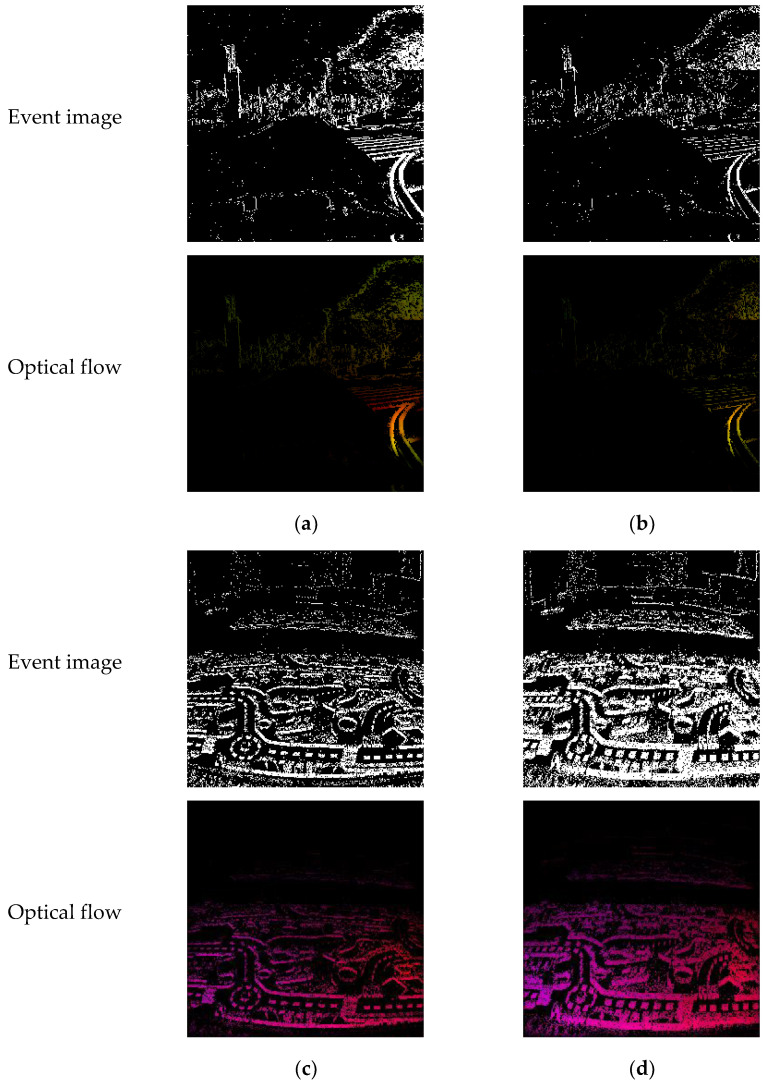
The optical flow prediction results after slicing by different methods. (**a**) and (**c**) do not have information loss or motion blur phenomenon by adaptive slicing compared with constant time interval; (**b**) information loss caused by constant time interval compared with adaptive slicing; (**d**) motion blur caused by constant time interval compared with adaptive slicing. (**a**) adaptive slicing, (no information loss); (**b**) constant time interval, (information loss); (**c**) adaptive slicing, (no motion blur); (**d**) constant time interval, (motion blur).

The optical flow prediction results after slicing by different methods are shown in Figure 8, which are from outdoor day1 and indoor flying1. In the data collection process, the camera motion speed changes, leading to the target information loss as shown in Figure 8b or motion blur phenomenon as shown in Figure 8d in the constant time interval method at some time, affecting the optical flow prediction accuracy compared with adaptive slicing as shown in Figure 8a,c.

## 4. Conclusions

In this paper, we construct an end-to-end spike neural network model for the event camera’s optical flow prediction more suitable for the discrete spatiotemporal event stream. Unlike the current neural network for optical flow prediction, we can directly handle the discrete spatiotemporal event stream output by the event camera, reducing the network computation. Compared with the convolutional neural network with the same network structure during hardware implementation, the proposed algorithm can save 99% more power consumption than the existing CNN algorithm, which is greatly beneficial to the hardware implementation of the event camera optical flow prediction. At the same time, the network is trained in the space domain and time domain by using spatial-temporal backpropagation in a self-monitoring way, which makes full use of the spatiotemporal information of spatiotemporal data flow and greatly improves the network performance. Moreover, to avoid motion blur or information loss, we adaptively slice the spatiotemporal data stream, and send event slices into the network, improving the accuracy of optical flow information. Finally, compared with other existing methods on the public dataset, the accuracy of optical flow information predicted by our method is not inferior to that of existing methods.

## Figures and Tables

**Table 1:** The quantitive results compared with the recent works [1,22,24,37] on event-based optical flow estimation.

dt = 1 Frame	Indoor Flying1		Indoor Flying2		Indoor Flying3		Outdoor Day1	
	AEE	%Outlier	AEE	%Outlier	AEE	%Outlier	AEE	%Outlier
Zhu et al. [24]	0.58	0.0	1.02	4.0	0.87	3.0	**0.32**	0.0
EV-FlowNet [22]	1.03	2.2	1.72	15.1	1.53	11.9	0.49	0.2
Spike-FlowNet [1]	0.84	0.0	1.28	7.0	1.11	4.6	0.49	0.0
STRN -FlowNet [37]	**0.57**	0.1	**0.79**	**1.6**	**0.72**	**1.3**	0.42	0.0
ours	0.76	**0.0**	1.13	6	0.95	4	0.45	**0.0**

**Table 2:** Analysis for operations and overall computational energy benefits compared with CNN.

	Indoor Flying1	Indoor Flying2	Indoor Flying3	Outdoor Day1
Spike Activity	0.38%	0.75%	0.62%	0.47%
Num. Operations Of SNN	0.37 × 10^8^	0.74 × 10^8^	0.60 × 10^8^	0.48 × 10^8^
Num. Operations Of CNN	7.89 × 10^9^			
Energy benefit	1088×	559×	671×	838×
Compute-energy Reduction	99.91%	99.82%	99.85%	99.88%

**Table 3:** Optical flow prediction results obtained by different slicing methods.

	Indoor Flying1	Indoor Flying2	Indoor Flying3	Outdoor Day1
Constant time interval	0.78	1.14	0.96	0.51
adaptive slicing	**0.76**	**1.13**	**0.95**	**0.45**

## Data Availability

Not applicable.
